# A New Perspective of Acupuncture: The Interaction among Three Networks Leads to Neutralization

**DOI:** 10.1155/2019/2326867

**Published:** 2019-02-24

**Authors:** Ning-cen Li, Ming-yue Li, Bo Chen, Yi Guo

**Affiliations:** ^1^Acupuncture and Moxibustion College of Tianjin University of Traditional Chinese Medicine, Tianjin, China; ^2^Acupuncture Research Center of Tianjin University of Traditional Chinese Medicine, Tianjin, China; ^3^Chinese Medicine College of Tianjin University of Traditional Chinese Medicine, Tianjin, China

## Abstract

Acupuncture has been used to treat multiple medical conditions, but whether the diverse effects of acupuncture are intrinsically linked and how they might be connected have yet to be determined. More and more researches have shown that acupuncture is a kind of nociceptive stimulus, which can cause inflammatory reaction in the sites of acupuncture and then further activate the nerve-endocrine-immune systems to cause the cascade amplification of the acupuncture effect. This review seeks to provide a comprehensive summary of the existing literature concerning the role of “acupoint-meridian-disease network” in various effects of acupuncture and suggest a novel notion that acupuncture may restore homeostasis under different pathological conditions by regulating this network, resulting in the activation of different reaction cascades in response to pathological injury. We think that acupuncture acts on acupoints, first activating the small network of acupoints (Acupoint Network). The information of acupuncture is amplified by cascade, and the nerve endocrine immune system (NEI) is activated through the large network of meridians (Meridian Network) of the body itself. The nerve-endocrine-immune system (NEI) further outputs the effect information to the target organ through multilevel and multisystems and finally acts on the disease network (Disease Network) to produce acupuncture effect.

## 1. Introduction

Acupuncture is an important part of traditional Chinese medicine, and significant efforts have been made to prove that acupuncture has the characteristics of easy operation, low price, outstanding efficacy, etc. [1]. According to the World Health Organization (WHO) traditional medicine strategy (2014-2023), acupuncture has been applied in 183 countries [2]. And in 2002, the WHO have released a report stated that 107 specific conditions may be suitable for acupuncture treatment [3]. A domestic study shows that after summing up clinical diseases and symptoms treated by modern acupuncture abroad, 110 diseases and symptoms were considered effective. And the disease spectrums were mainly distributed in muscle, bones, and connective tissue [4]. As an experience-based medicine with a long history, the clinical efficacy of acupuncture has been accepted all over the world. It contributed enormously to the health of the people of China and world. National Institute of Health of USA (NIH) has admitted to acupuncture treatment of chronic pain and other pathological conditions [5].

With the clinical efficacy of acupuncture widely recognized in the world, basic experimental research is an important basis for clinical practice guidelines, and it can better promote the development of acupuncture in the world. Many scholars at home and abroad have begun to carry out basic experiments to clarify the mechanism of acupuncture and moxibustion, finding some effective biological molecules in cerebrospinal fluid, serum, organs, and acupoint tissue. Acupuncture can affect the expression of bioactive genes and proteins in physiological and pathological [6]. In recent years, researches have focused on analgesic and anti-inflammatory which have already made some progresses. Acupuncture can promote the release of anti-inflammatory and analgesic substances (opioid peptides, adenosine, dopamine, and endogenous Cannabinoids [7–9]) and inhibit the release of proinflammatory factors (5-hydroxytryptamine, histamine, substance P, nerve growth factor, CGRP, and TRPV1 [10–12]) to produce analgesic effect. Most basic studies focus on the linear correlation between “intervention and effect”, but ignore the comprehensive network regulatory effects produced by acupuncture as complex interventions. Acupuncture can affect the function of multiple organs or systems at the same time. The regulation of acupuncture on the organ function is achieved through the integrated regulation of the system that the organ belongs to and even the whole body [13]. Acupuncture is a complex physical stimulation to transfer information from the acupoints to the target organ by meridians, including the release of chemical substances, the activation of numerous cells, and the related nerve excitation. Acupuncture activates the peripheral nerves and transfers the input signals through various neural pathways to the brain and spinal cord. The neurofunctions of the peripheral and spinal levels play some physiological roles conversely, such as nerve protection and analgesia. This determines that the effect of acupuncture is not the result of single factor, but the complex interaction of multiple factors, levels, and systems. However, the network relationships among those factors have not been specifically elaborated yet.

In the organism, the “neutralization” state refers to the best homeostasis of the movement of living substances in healthy individuals. Acupuncture treatment of diseases is mainly through the adjustment of the internal environment of the body, so as to achieve the purpose of curing the disease by restoring the “Yin and Yang in harmonious” state. In order to better understand the basic mechanism and scientific basis of acupuncture and moxibustion, we think that acupuncture acts on acupoints, first activating the small network of acupoints (Acupoint Network). The information of acupuncture is amplified by cascade, and the nerve endocrine immune system (NEI) is activated through the large network of meridians (Meridian Network) of the body itself. The nerve-endocrine-immune system (NEI) further outputs the effect information to the target organ through multilevel and multisystems and finally acts on the disease network (Disease Network) to produce acupuncture effect.

## 2. The Acupoint Network Is the Source of Acupuncture Effect and the Trigger Point of NEI Network

### 2.1. The Structural Basis of the Acupoint Network

The complex construction of acupoints is the structural basis of the Acupoint Network. Acupoint is the basis of the effect of acupuncture which plays an important role in the acupuncture treatment of diseases. Acupoint is a stereoscopic structure composed of skin, subcutaneous tissue, nerve [7, 14], blood vessel [15], lymph, muscle [16], and connective tissue. There are abundant dense receptors, peripheral nerve endings, nerve bundles, nerve plexus, capillaries, and others in the acupoint area, which are more sensitive to the stimulation like acupuncture, electroacupuncture (EA), laser acupuncture, and so on [13].

### 2.2. The Biological Basis of the Acupoint Network

The specific changes in the local microenvironment caused by acupuncture are the biological basis of the Acupoint Network. Morphological structure studies were analyzed from the perspective of the anatomical structure. Luo reported that a strip-like compound structure of mast cells, blood vessel, and nerve network is observed in acupoint area, indicating that interrelation and interaction among blood-vessel network, nerve network, and immune network are considered to be the key to reveal the mechanism of acupuncture treatment and the essence of meridians [17]. Zhang et al. define the collection of the activated neural and neuroactive components surrounding the inserted needle as a neural acupuncture unit (NAU), who first constructs the acupuncture nerve regulating network [18]. Cui et al. used neural tracing technique as an effective approach to reveal the sensory and motor innervations of PC8 in order to explore the neuroanatomical characteristics of acupuncture points [19]. Nanna Goldman found that a neuromodulator with antinociceptive properties “adenosine” was released during acupuncture and benefit analgesic effect of acupuncture [8]. Several studies have shown that local adenosine triphosphate (ATP) is one of the key factors for producing acupuncture analgesia [20]. And favorable regulation of acupuncture on the abnormal functional activities of some viscera often accompanies an increase of ATP content or ATPase activity [21]. Mast cells are found abundant in acupoints [22, 23]. Under the pressure, TRPV2 proteins open to induce mast cell activation and degranulation, further causing increases in histamine and adenosine concentrations in local tissue [24]. Wei Yao et al. [25] further confirmed the role of local mast cell, histamine, and other neurotransmitters in the initiation of acupuncture effect, while the mast cell–nerve axis in the human intestine can provide anatomical basis for the realization of information transmission [26]. Except mast cell-released substances, many immune mediators like local tissue-released NA, nitric oxide (NO), tumor-related factors, and so on, are heavily involved in the determination of electrical properties of acupoints and meridians [27, 28]. Naloxone, opioid receptor antagonist, *β*-endorphin antagonist, and corticotropin-releasing factor antagonist also have effects on electroacupuncture of acute and chronic inflammatory pain [29, 30]. Some researchers have shown that electroacupuncture can increase the quantity of CGRP and SP in peripheral tissues and in blood circulation [31]. Acupuncture can cause specific responses in the local microenvironment of acupoints, such as the activation of cell function, the release of chemical substances, and the excitation of the afferent nerve. The interaction among them may trigger and successively amplify acupuncture signal to produce a whole regulating effect by directly or indirectly acting on the corresponding receptors on the surface of the afferent nerve.

In addition, the development of genomics and proteomics provides help for research. Xu et al. [32] use proteomic techniques to find that there are 25 significant proteins in the acupoints of normal rats after acupuncture. They maintain internal homeostasis via regulation of the local stimulus response, biomolecule function balance, and energy metabolism which maybe contribute to the therapeutic effects of acupuncture. Ji-Yeun Park [33] used gene chip technology to find that there are at least 7 neuroimmunological pathways involved in the analgesic and anti-inflammatory process of acupuncture at GB34 for inflammatory pain model, and ERK activation in the skin layer which contributes to the analgesic effect.

**Figure 1:**
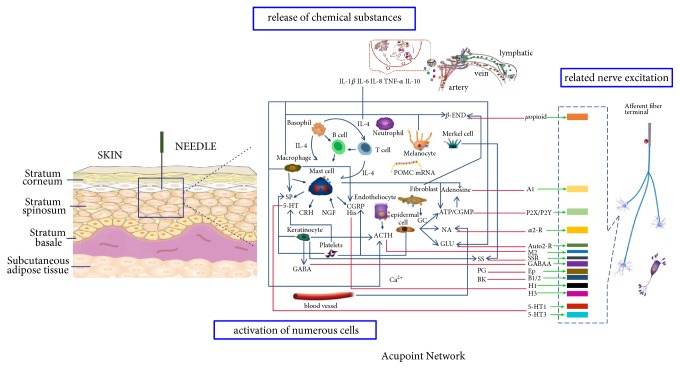
The diagrammatic sketch of Acupoint Network. Acupuncture can cause specific responses in the local microenvironment of acupoints, such as the activation of cell function, the release of chemical substances, and the excitation of the afferent nerve. The interaction among them may trigger and successively amplify acupuncture signal to produce a whole regulating effect.

Based on the comprehensive analysis of the research progress at home and abroad combined with the results of our laboratory, we put forward the concept of “acupuncture point promoter” [34]. It is believed that acupuncture can cause specific response changes in the local microenvironment of acupoints, involving many aspects like divine meridian, endocrine, immunity, and so on [35]. They constitute the initiation network of the acupuncture effect. Therefore, we further proposed the concept of “Acupoint Network”.  Figure 1 shows the formation process of “Acupoint Network”, and Table 1 introduces some major mediators involved in the modulation of this network. (Table 1, Figure 1)

## 3. The Meridian Network Regulation System Based on Neuroendocrine-Immune Network Is the Biological Basis and Core Link for Maintaining Homeostasis

### 3.1. The Meridian Based on Acupoints Itself Is a Complex Network Linking the Whole Body

The meridian system consists of meridians (Jing Mai) and collaterals (Luo Mai). Meridians are pathways in which the qi and blood circulate and through which the viscera and limbs are connected, allowing the upper-lower and interior-exterior portions of the body to communicate. They link together to unify all the viscera, orifices, muscles, organs, skin, and bones in the human body into an organic whole [44].

The meridian system is a complex network connected by 361 acupoints. Acupuncture points can cause the conduction of qi in the meridians and collaterals. It is found that when the acupoint is stimulated, arrival of qi from the acupoint and along the meridian would appear, that is, propagated sensation along the channels [13]. The Meridian Network has a wide range of physiological functions, including the nervous system, endocrine system, and immune system. The connection between acupoints and effects is mediated by the Meridian Network based on the “neuroendocrine-immune circuit” [45]. The specific changes in the local microenvironment of acupoints are closely related to the regulation of the NEI network, thus activating the regulation system of Meridian Network. The synaptic junction between mast cells and nerve endings was confirmed by immunohistochemistry and fluorescence microscopy, which provided a histoanatomical basis for the interaction between Acupoint Network and Meridian Network [46, 47]. Preliminary studies have shown that mast cell-nerve interaction can balance the stability of local microenvironment. External stimulation can lead to mast cell activation, release many chemicals, and act on adjacent nerves. Neural endings release transmitters and regulate the function of mast cells, thus maintaining the stability of Acupoint and Meridian Network [48].

### 3.2. The Relationship between the Meridian and Nervous System

The Huang Di Nei Jing takes the governor vessel (Du Mai) and bladder meridian (PangGuang Jing) as an example to describe the anatomical relationship between the meridians and brain and suggest that the connecting structure between the visual organs and the brain may be the optic nerve. The meridians are closely related to the central nervous system. Zhang Dong et al. found there were similar characteristics between the temperature response in the cortex showing by cortical infrared thermography (CIT) after EA and the activity of cortical nerve cells after stimulation [49]. Tian et al. found acupuncture at GB37 and GB34 can stimulate the visual cortex of bilateral occipital lobe, while acupuncture at ST36 and ST32 can stimulate the hypothalamus, hippocampus, and frontal gyrus by BOLD-based fMRI technique [50]. Lee B. et al. observed the behavioral effect of the acupuncture can originate from the modulation of the neuronal mechanism in the central dopaminergic system [51]. The meridians are also related to the peripheral nervous system. The anatomy teacher of Shanghai No. 1 medical college had autopsied and observed that there was nerve distribution in 57 acupoints which were in line with the meridians to a certain extent [52]. Based on the technique of infrared image information, the inherent infrared radiant tracks over the body surface were successfully displayed by Hu X. under the natural conditions. Most of these linear radiant tracks were coincided with that of the classical Fourteen Meridians [53]. Liu et al. used the high pressure liquid chromatography to find the distribution lines of sympathetic substances in skin after acupuncture. And based on the sympathetic nerve network of the pili muscle, they put forward this line as the transmission line of acupuncture signals [54]. Li et al. suggested that both mechanical and EA stimulation of ST36 at a certain intensity can produce a change of discharges of the afferent nerve, preliminarily clarifying the immediate effect of peripheral nerve after acupuncture [55].

Neurotransmitters are specific chemicals that act as “messengers” in synaptic transmission and play an important role in the central and peripheral nervous systems. Some scholars have found that neurotransmitters are enriched in neurotransmitters at most meridians [56]. Ma et al. took heart channel for instance to study the connection pathway between channels and viscera by immunohistochemical ABC method and found the presence of neuropeptides in the meridians [57]. All the above provide evidence for the close relationship between meridians and nervous system.

### 3.3. The Relationship between the Meridian and Endocrine System

Depending on the pituitary, hypothalamic-pituitary-adrenal axis, hypothalamic-pituitary-thyroid axis, and hypothalamic-pituitary-gonadal axis, the relationship is mainly manifested in the release of endocrine hormones after acupuncture. The nervous system plays a leading role in this process, as all of effects are generated by the neural arc.

Traditional Chinese Medicine theory holds that women's menstruation and amenorrhea are related to conception vessel (Ren Mai) and thoroughfare vessel (Chong Mai) [58]. Modern studies believe that the menstrual cycle is regulated by the interaction among the reproductive hormones of the hypothalamus, pituitary, and ovary. If there is a disorder, it can lead to some gynecological diseases like polycystic ovary syndrome [59]. Conception vessel and thoroughfare vessel are related to the regulation of endocrine system. Wang et al. injected Evans blue into inflammatory rats through the caudal vein and then found that exudative points mainly distributed at spleen meridian, stomach meridian, conception vessel, thoroughfare vessel, and the spinal cord segment of the acupoints are consistent with the one that dominates ovarian function [60].

### 3.4. The Relationship between the Meridian and Immune System

The most direct connection between the meridian and immune system is the morphological relationship of lymphatic system. Some scholars found that the three yin meridians of foot and lymphatic vessels have similar routes in X-ray [37]; then this similarity is described meticulously in the anatomic atlases [61]. Many experiments suggesting that the function of physical immune active cells like T lymphocytes was increased by EA on stomach meridian and might play an active role in increasing physical resistance [62]. Researchers found the number of mast cells around the acupoints was significantly higher than that of nonacupoints, and the metabolism was strong. Acupuncture stimulates mast cell degranulation and produces chemokines such as histamine and eosinophils which activate the immune response [63].

### 3.5. Nerve-Endocrine-Immune Network Is Constructed through Common Signal Molecules

Besedovsky first proposed the concept of “neuroendocrine-immune (NEI) network” in 1977 and believed that three systems did not exist separately, but correlated and interacted with each other [64]. Modern research shows that the three systems of nerve, endocrine, and immunity are closely linked through the universal language of them [65]. Paracrine signals like lymphokines released by synapses can affect the functions of immune cells, and neuroendocrine immunologic substances can coexist in the same nerve cell, which establish the material basis for the relationship among nerves, endocrine, and immune system [66]. Rafael Torres-Rosas reported that electroacupuncture at the sciatic nerve controls systemic inflammation by inducing vagal activation of aromatic l-amino acid decarboxylase, which can lead to the production of dopamine in the adrenal medulla [67]. Cytokines like IL-1, IL-2, IL-6, INFs, and TNF can regulate the neuroendocrine system [68]. Glucocorticoids (GC), growth hormone (GH) and opioid peptides have a regulatory effect on the immune system [38, 69]. Functions as a neuropeptide in the central nervous system, ghrelin, can promote the release of somatotropin and stimulate the secretion of hormones and other neuropeptides like neuropeptide Y gene and hormone [70, 71]. The connection among the three systems is often a two-way adjustment between them, while any change in one system will directly or indirectly affect the other systems to further influence the body. The overall regulation effect of acupuncture cannot be without the regulation of acupuncture on NEI network. A large number of acupuncture experimental studies also show that the production of acupuncture is related to the participation of three systems [35]. Zhou Dan et al. took the mast cell as the breakthrough point, indicating that acupuncture is a kind of nociceptive stimulus, which can cause inflammatory reaction in the sites of acupuncture [72]. As one of the initial factors of acupuncture effect, the inflammatory reaction further activates the nerve-endocrine-immune network to cause the cascade amplification of the acupuncture effect. The interrelation and interaction among nerve network, endocrine network, and immune network are considered to be the key to reveal the essence of meridians. Luo et al. thought that the structure of mast cells, blood vessel, and nerve network is an interactive system, as a core structure of acupoint; it is a significant pivot to produce and transport substances, energy, and information simultaneously [73].

As part of acupuncture stimulation, skin and central nervous system come from neuroectodermal layer together. The complex distribution of nerves in the skin and the expression of many hormones and their receptors on the skin, hair follicles, and secretory glands suggest that there is a certain connection between the skin and neuroendocrine system [74, 75]. Some researchers call it the largest “neuroendocrine organ” [76]. After isolating the hair follicles from the HPA axis and nerve vessels of the central system, a series of reactions similar to those of the central HPA axis system were produced when corticotropin-releasing hormone (CRH) was used to stimulate the cultured hair follicles in vitro. Forming cells, melanocytes, fibroblasts, endothelial cells, and mast cells have been proved to synthesize and secrete CRH [42, 77]. CRH, as the most upstream factor of skin HPA axis, can induce HPA stress response on the one hand and can bind to CRHR receptors in the skin and initiate a variety of signaling pathways to function on the other hand [43].

A large number of studies have confirmed that nerve, endocrine, and immune systems have formed an interrelated and interdependent network which can regulate activities of the body. The local microenvironment changes after acupuncture stimulate the network control system of the Meridian Network, and the effective information of acupuncture is further carried out to act on target organ “disease network” in cascaded way.

## 4. The Disease Network Is the Key to Understand and Treat Diseases

### 4.1. The Concept of Disease Network

Huang Di Nei Jing is the earliest work on the names, etiology, location, pathogenesis, and main symptoms of diseases. WHO released an open letter of a new definition of health: human equilibrium in nature, accepted spirituality, and adaptation. That is to say, health is a physically, psychologically, socially adaptable, and morally well situation [78]. A disease is often caused by multiple factors, involving the dysfunction of polygenes and multiple targets. The interaction of various factors in the process of disease formation constitutes a “disease network”. Therefore, single target therapy is difficult to improve all symptoms. For example, when we searched “rheumatoid arthritis” in the human disease database (http://www.genecards.org/), we could find that there are 1872 genes associated with the disease.

In 2015, Obama proposed a “precision medicine” program, which aims to establish a complete network of disease knowledge by searching for detailed information about the individual genome, proteome, metabolome, and transcriptome, so as to conduct individualized treatment and evaluate the possibility of disease variation. The development of high-throughput biotechnology provides a large number of data for disease research, while bionetworks represented by the gene regulatory network and protein interaction network provide good data support for the study of complex diseases.

Li Shao [79] proposed a TCM-derived novel therapeutic concept “network target and herbal combination” as an emerging network pharmacology approach to unveil holistic medicine. The advantage of TCM in the treatment of rheumatoid arthritis is that it can be treated as a whole by regulating the NEI network on the basis of the network prediction of the disease gene. Similarly, the overall regulation effect of acupuncture is also inseparable from the adjustment of the NEI network by acupuncture. The effect of acupuncture is not the result of a single factor, but a complex process involving multiple factors, levels, and systems.

### 4.2. The Disease Network Can Be Reflected through the Meridian Network to the Acupoint Network

The meridians can communicate inside and outside, upper and lower, so the functional state of the body can also be reflected through the acupuncture points. When the body is in a pathological state, the disease network changes to be reacted to the points through the Meridian Network. The acupoint “A Shi” which was first seen in the Bèijí Qiānjīn Yàofāng (Important Formulas Worth a Thousand Gold Pieces for Emergency) is the best representative of acupoints with some reactions like acid, hemp, swelling, pain, weight or spot, color changes, hard changes, and so on. When some disease occurs, a part of the human body is blocked by qi and blood, causing the local and temporary accumulation, thus appearing a phenomenon called “A Shi”, and when the disease is relieved, the phenomenon disappears [80]. Two main therapeutic effects that acupuncture can bring about are the local effect and the distant effect. The foundation of meridian system by ancient people heavily emphasized the local lesion and effects of acupoints on the limbs. If the acupoints on the limbs only had the local effects, the “tenderness as acupoint” would be enough for acupuncture treatment and there would not develop the meridian system.

## 5. Discussion

It is believed that the living environment of the organism is composed of constantly changing external environment and relatively stable internal environment [81]. The stability of the internal environment is a necessary condition for maintaining the physiological functions of human life, tissue, and organ. Under the regulation of nerve and body fluid, the body can maintain the relative stability of the internal environment of the normal metabolism in the body, that is, “homeostasis”. But when the environmental changes exceed the regulation that we can bear, we need external medical means to solve this problem.

Modern medical methods often regulate the fluid and electrolyte imbalance by using the corresponding agonist antagonists in western medicine or infusion; however, we cannot guarantee that this simple “point to point” or “point to system” mode of action is detrimental to human self-regulation.

Modern research believes that as an open and complex giant system, organisms are formed by the interconnection and integration of numerous networks of different sizes, not a superposition of simple systems [82]. Acupuncture, as the original medical science of the Chinese nation, reveals the laws of human health and the occurrence/development of diseases from a macroscopic, systematic, and overall perspective. TCM is a balanced medicine. While maintaining the health of the human body, it is further to pursue a new balanced environment according to the pathological state, rather than to overemphasize the removal of the cause. All the purposes of the treatment are the balance of yin and Yang. The core of acupuncture treatment is also regulating yin and Yang. The mode of action based on the linkage of three networks is the biological basis of maintaining the body's stability to treat the disease. It is also an important reason for the wide range of clinical application and remarkable curative effect of acupuncture. The domestic research has found more than 500 kinds of suitable diseases of acupuncture [83].

**Figure 2:**
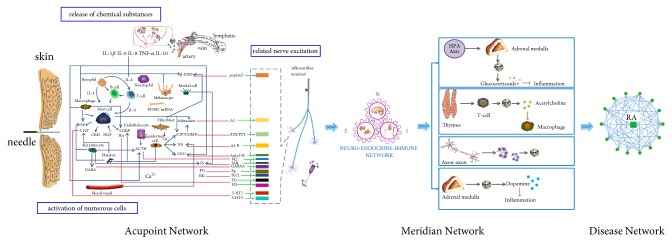
The diagrammatic sketch of triple-network linkage. (Acupuncture first activates the small network of acupoint microenvironment, enabling acupuncture information to be initiated and cascaded locally. Then it mobilizes the Meridian Network controlled by NEI and finally acupuncture effect information is output to the target organs and produce acupuncture efficacy.)

How does acupuncture regulate the body to neutralization? We first proposed the “three-network linkage” theory that acupuncture initiates the Acupoint Network, mobilizing the body network of the Meridian Network, adjusting the Disease Network, and finally achieving yin-yang coordination and the state of neutralization.  Figure 2 shows “Acupoint-Meridian-Disease network”. Specifically, as a traumatic physical stimulation, acupuncture first activates the small network of acupoint micro-environment, enabling acupuncture information to be initiated and cascaded locally. Then it mobilizes the Meridian Network controlled by NEI, finally acupuncture effect information is output to the target organs and produce acupuncture efficacy. Triple-network linkage corrects the imbalance of the disease and restores the internal homeostasis, further achieves “neutralization”. When the body is in a pathological state, the disease network changes, and the NEI network of the body can also be reflected to the local Acupoint Network, so that some positive reactions such as tenderness, trabs, and tubercles and tenderness are generated which can play an “alert” role for patients and doctors.

## 6. Development Tendency and Prospect

The action pattern of acupuncture is different from that of the western medicine. Its mutual regulation effect can also be described by “cross talk”. It is of great significance to reveal the general law of acupuncture and improve the curative effect of by studying the related network relations of these factors and carrying out the research on the characteristics of acupuncture from the multiple systems.

The mechanisms triggering acupuncture effects have appealed intensive attention on. Acupuncture is the systematical medicine that emphasizes on general regulations, and continuous development of omics techniques has enabled to explain network regulatory effects. The major elements of effects of acupuncture therapy include promoter, microenvironment changes in acupoints, signaling pathways, responders, and effectors. That is, the triple-network linkage made of Acupoint Network, Meridian Network, and Disease Network. Acupuncture has its immediate and subsequent effects. Immediate effects are always related to the nerve systems, while subsequent effects are related to cytokines, hormones and other signals in body fluids. After generally understanding the mechanisms triggering acupuncture effects and combining the three networks, new regulatory patterns can be explored to promote the transformation of basic studies and improve clinical application level.

## Figures and Tables

**Table 1:** Major mediators involved in the modulation of “Acupoint Network”.

Mediators	Cells releasing neuroactive mediators	Receptors on afferent fiber terminals	Reference
5-Hydroxytryptamine	Platelets, mast cells	5-HT_3_ receptor	[10]
		5-HT_1_ receptor	
adenosine	Mast cells	A_1_ receptor	[8]
histamine	Mast cells	H_3_ receptor	[11]
		H_1_ receptor	
substance P	Keratinocytes, platelets,	-	[12]
	mast cells, fibroblasts, and macrophages		
dopamine		DA2R	[9]
Calcitonin	Macrophages,	-	[31]
gene-related peptide (CGRP)	T cells, epithelial cells		
Glutamate	Skin epithelial cells and macrophage	-	[36]
ATP/cGMP	Epidermal cells, Mast cells	P2X, P2Y receptor	[20]
IL-1*β*, IL-6, IL-8, and TNF-*α*		-	[37]
Glucocorticoids (GC)	tissues	glucocorticoid receptor (GR)	[36, 38]
Growth hormone (GH)	Eosinophils of anterior pituitary	GH receptor	[36, 38]
Noradrenaline (NA)	sympathetic nerve varicosities	*α*2 Receptors	[27]
Nitric Oxide (NO)	tissues	-	[28]
Bradykinin	tissues	B_1/2_ receptors	[39]
Somatostatin (SS)	Merkel cells, keratinocytes	SS receptors	[40]
*γ*-aminobutyric acid (GABA)	Macrophages and lymphocytes	GABA_A_ receptors	[41]
*β*-endorphin	Keratinocytes, melanocytes, dermal fibroblasts, leukocytes	*μ*-opiate receptors	[29, 30]
CRH	hypothalamus	CRH receptor	[42, 43]
ERK1/2	fibroblast and keratinocyte	-	[33]

## Data Availability

The data and materials sources in this paper are public.

## Abbreviations

NEI: Nerve endocrine immune system
WHO: World Health Organization
NIH: National Institute of Health of USA
5-HT: 5-Hydroxytryptamine
His: Histamine
SP: Substance P
NGF: Nerve growth factor
CGRP: Calcitonin gene related peptide
TRPV1: Transient receptor potential cation channel subfamily V member 1
NAU: A neural acupuncture unit
ATP: Adenosine triphosphate
NO: Nitric oxide
GC: Glucocorticoids
GH: Growth hormone
NA: Noradrenaline
NO: Nitric Oxide
SS: Somatostatin
GABA: *γ*-aminobutyric acid
CIT: Cortical infrared thermography
HPA: Hypothalamic-pituitary-adrenal axis
CRH: Corticotropin-releasing hormone
ERK: Extracellular regulated protein kinases.
